# Supplementary material for: Cellular In Vitro Responses Induced by Human Mesenchymal Stem/Stromal Cell-Derived Extracellular Vesicles Obtained from Suspension Culture
Source: Int J Mol Sci. 2024 Jul 11;25(14):7605. doi: 10.3390/ijms25147605 (PMC11277484; doi:10.3390/ijms25147605)
Supplement: Supplementary file 1 [file ijms-25-07605-s001.zip › ijms-3072667-supplementary.pdf]

International Journal of
Molecular Sciences

MDPI

Supplementary data

Cellular In Vitro Responses Induced by Human Mesenchymal Stem/Stromal Cell-Derived Extracellular Vesicles Obtained from Suspension Culture

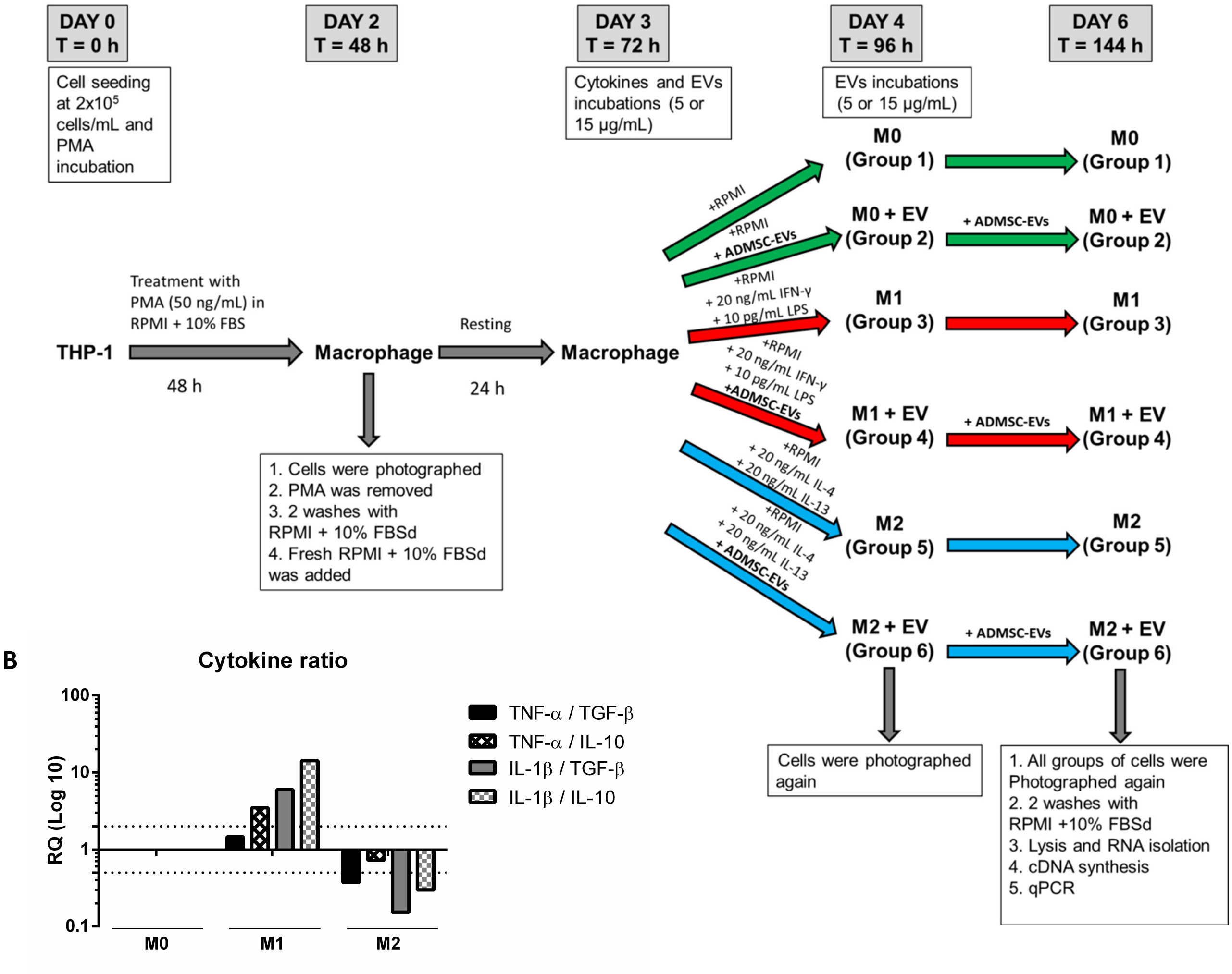

Figure S1. Macrophage polarization assay scheme and functional characterization of the model. (A) Macrophages groups were incubated with cytokines and with or without ADMSC-EVs at day 3 as following: (group 1) unstimulated control M0-like macrophages; (group 2) M0-like + ADMSC-EVs; (group 3) control M1-like macrophages; (group 4) M1-like macrophages + ADMSC-EVs; (group 5) control M2-like macrophages; (group 6) M2-like macrophages + ADMSC-EVs. At day 3 and 4, groups treated with ADMSC-EVs (2, 4 and 6) received two subsequential doses of 5 or 15 µg of extracellular vesicles per well at day 3 and day 4, totalizing 10 or 30 µg/mL. Groups 3 and 4 of pro-inflammatory M1 macrophages were treated with 20 ng/mL IFN-γ and 10 pg/mL LPS at day 3. Groups 5 and 6 of anti-inflammatory M2 macrophages were treated at day 3 with 20 ng/mL human IL-4 and 20 ng/mL IL-13. All groups were photographed at day 2, 4 and 6. At day 6 cells were lysed and RNA of each group was isolated for cDNA synthesis and qPCR. (B) Ratios of cytokines expression reveal different balances of pro-inflammatory and anti-inflammatory components among M0, M1-like and M2-like macrophages. The graph represents the experimental standardization without the use of EVs. Relative gene expression quantification (RQ) was calculated using the $-2^{\Delta\Delta CT}$ method.

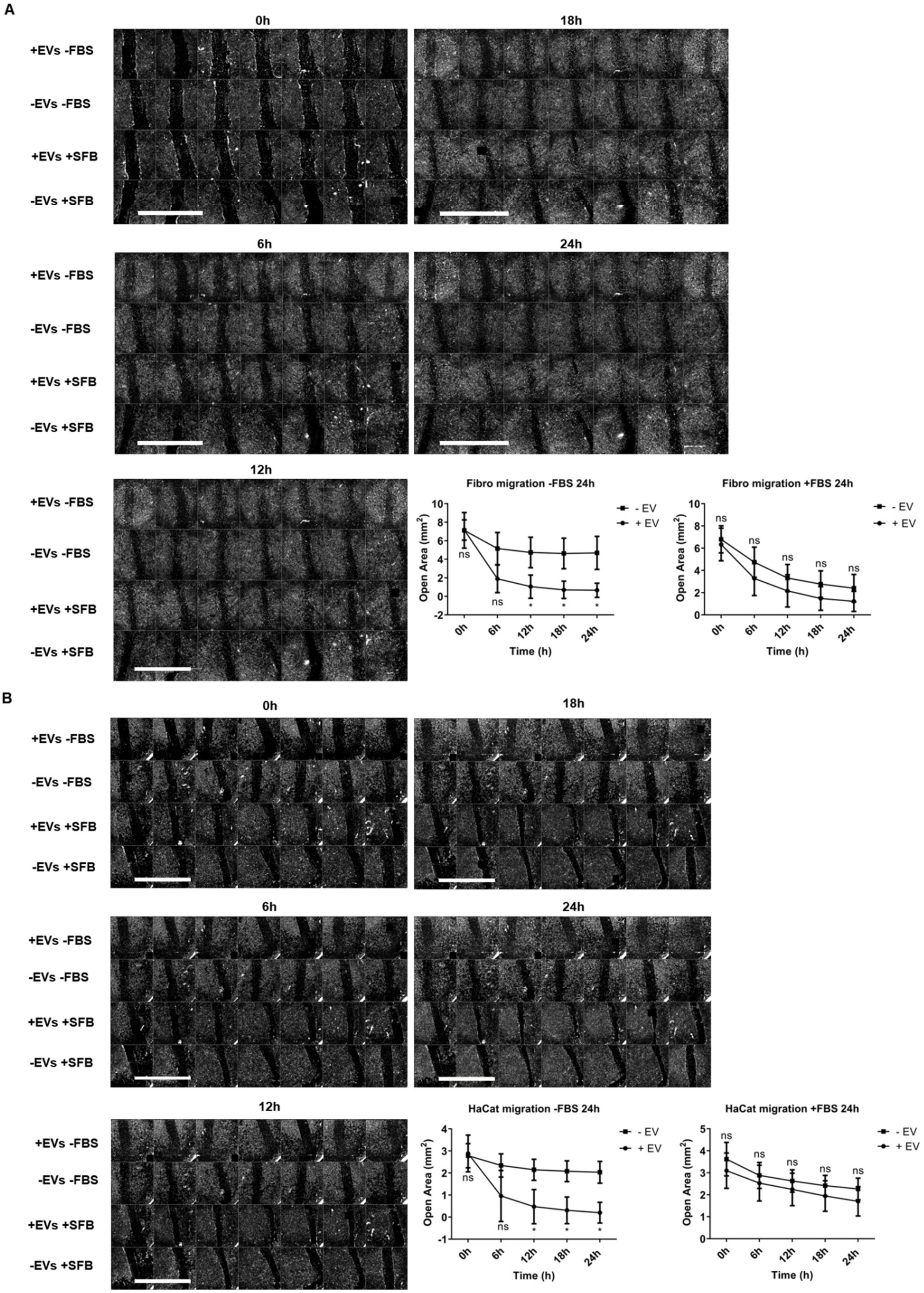

Figure S2. ADMSC-EVs induced migration in NHDF-1 (A) and HaCat (B). (A) Representative images of NHDF-1 migration in 0, 6, 12, 18 and 24 h and graphs of scratch/wound closure in 0, 6, 12,

18 and 24 h without or with starvation (FBS depletion) treated (+) or not (-) with 80 ng/μL ADMSC-EVs (data from 3 independent assays with nonoplicates, 9 wells for each condition). (B) Representative images of HaCat migration in 0, 6, 12, 18 and 24 h and graphs of scratch/wound closure in 0, 6, 12, 18 and 24h without or with FBS supplementation treated (+) or not (-) with 80 ng/μL ADMSC-EVs (data from 3 independent assays, with nonoplicates, 9 wells for each condition). For both cells, graphs show that in depletion of FBS, the treatment with ADMSC-EVs induces faster wound closure in comparison with non-treated cells. Also, for NHDF-1 and HaCat, the supplementation with FBS surpasses the effect of ADMSC-EVs on cell migration. For comparison of two groups, it was performed first a paired Student t-test, then differences among groups were evaluated by two-way ANOVA with Geisser-Greenhouse correction, and 95% confidence intervals were calculated. Differences were significant at *p <0.05 and **p < 0.01. Scale bar = 5 mm.

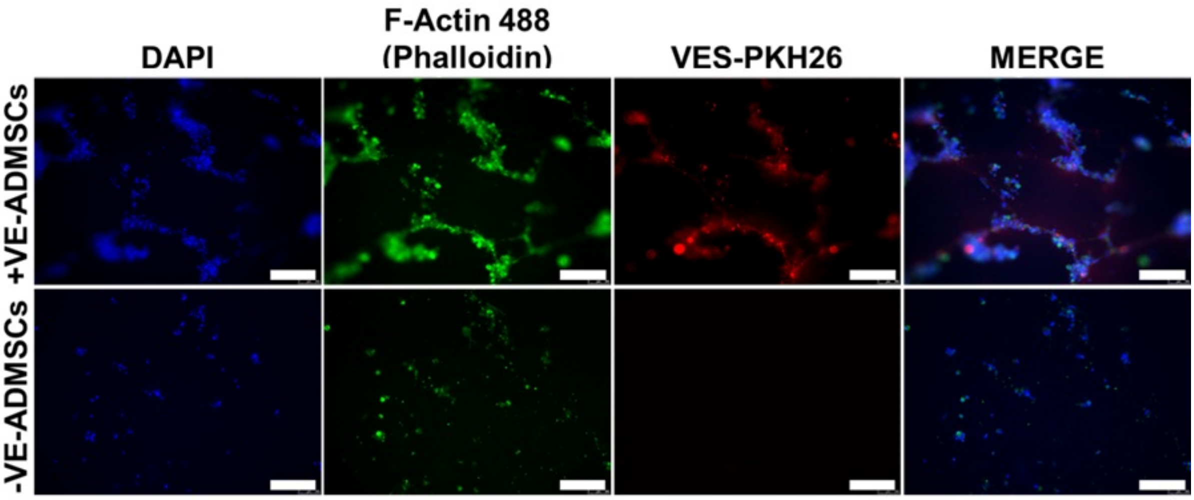

Figure S3. Immunofluorescence of HUVEC angiogenesis negative control. HUVEC cells were treated (+) or not (-) with 80 ng/μL ADMSC-EVs (EVs stained with PKH26 red) for 24 h and maintained in EBM medium depleted of supplements and FBS. Nuclei were stained with DAPI (blue). Cytoskeletons of cells were stained with phalloidin 488 (green). In cells treated with ADMSC-EVs it is possible to see that vessels are formed, but not totally connected nor closed. Scale bar = 50 μm.

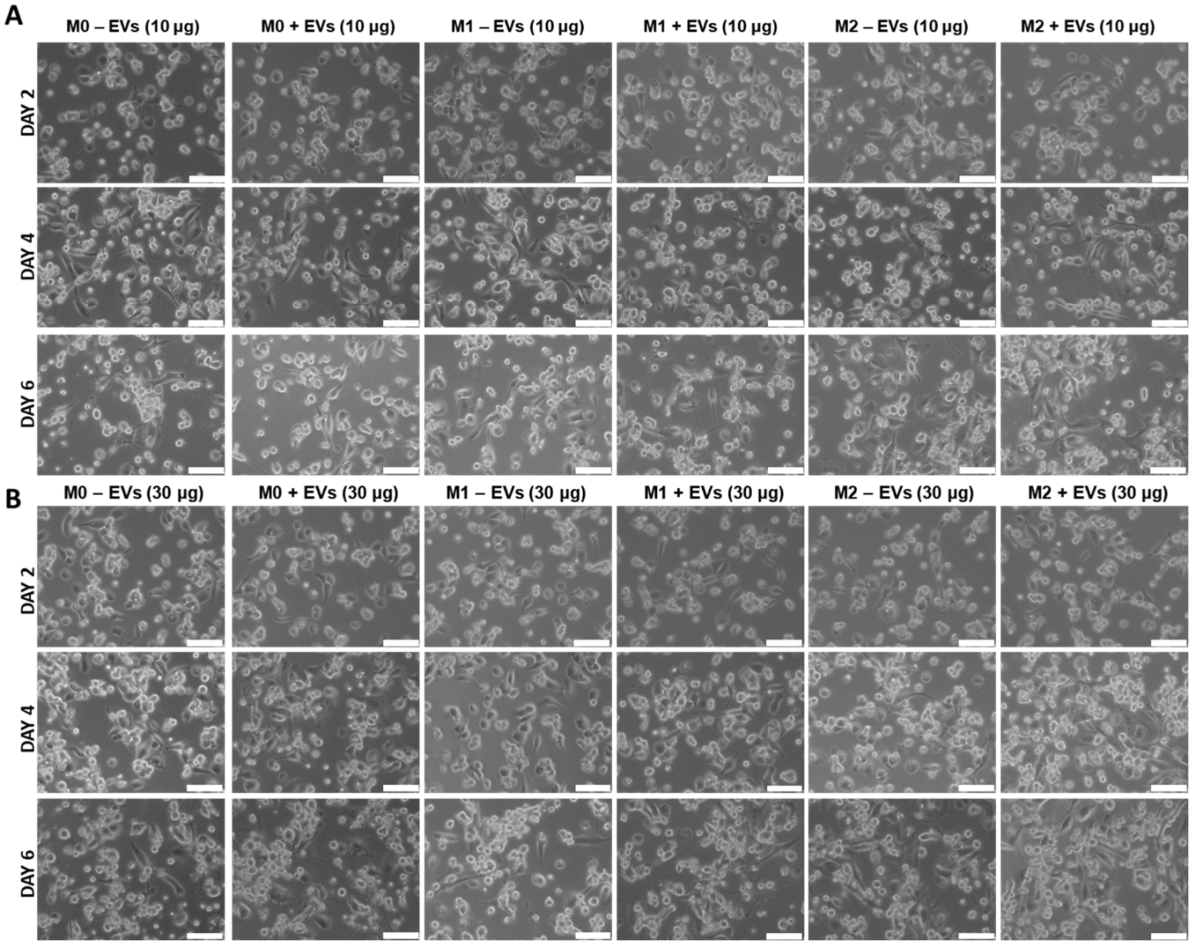

Figure S4. Images of M0, M1 and M2 macrophages treated or not with ADMSC-EVs. (A, B) Morphology of macrophages derived from monocytes THP-1 in days 2 (48h), 4 (96h) and 6 (144h), groups M0, M1 (differentiated with 20 ng/mL IFN-γ and 10 pg/mL LPS) or M2 (differentiated with 20 ng/mL IL-4 and IL-13) were treated (+) or not (-) with 10 µg/mL (A) or 30 µg/mL (B) ADMSC-EVs. Scale bar = 100 µm.

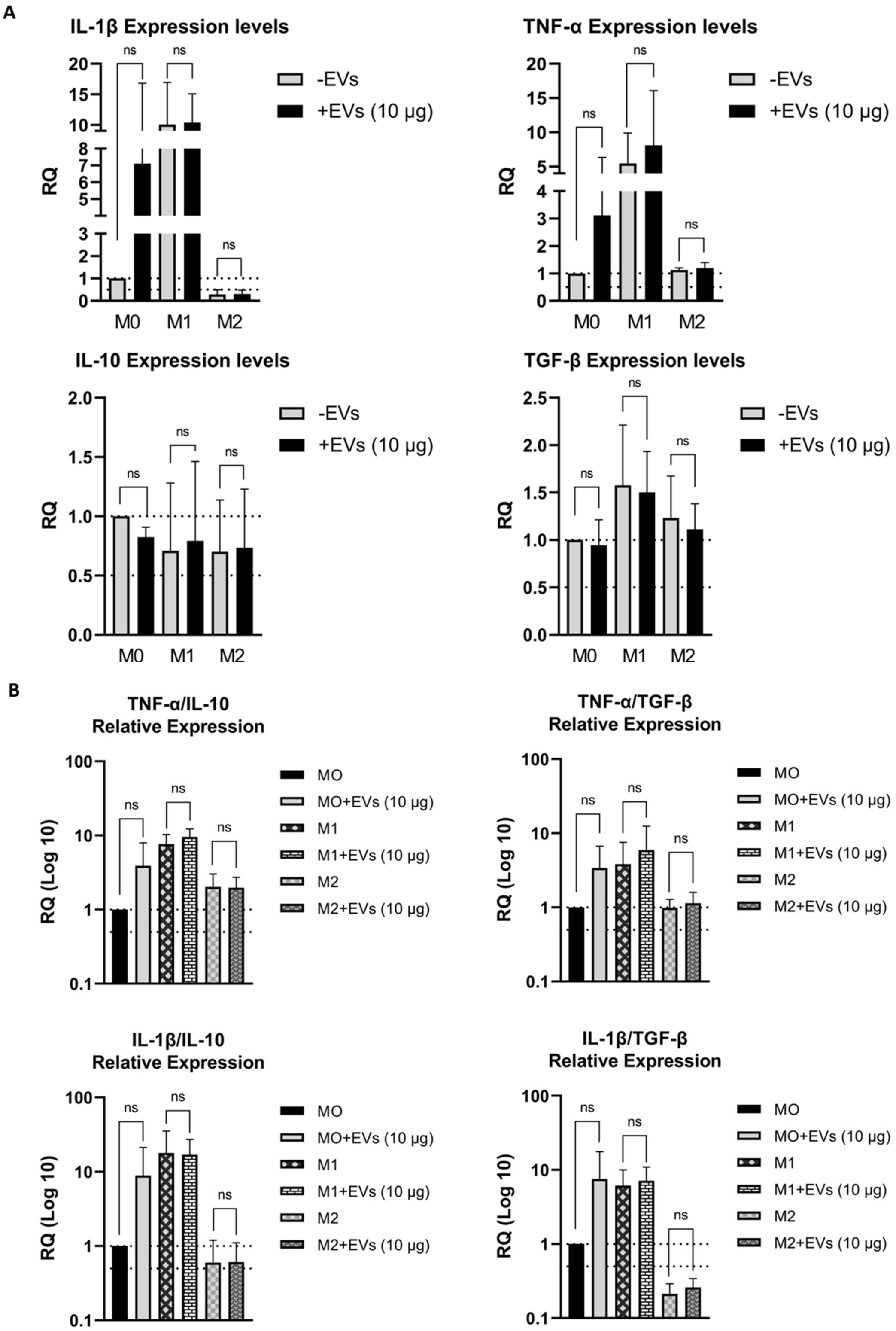

Figure S5. Lower concentration of ADMSC-EVs did not induce cytokines expression in all groups of macrophages. Graphs of cytokines expression on M0, M1-like and M2-like macrophages treated (+) or not (-) with 10 μg/mL (A) ADMSC-EVs. Graphs of ratios between pro-inflammatory and anti-inflammatory cytokines expression in M0, M1 and M2 macrophages treated (+) or not (-) with 10 μg/mL (B) ADMSC-EVs. Data obtained from 3 independent assays with triplicates each. Relative gene expression quantification (RQ) was calculated using the $-2^{\Delta\Delta CT}$ method. A two-way ANOVA with Šidák correction was used for comparisons between cells treated and non-treated with EVs, within a confidence interval of 95%. Differences were considered statistically significant at $^{**}p < 0.01$ and $^{***}p < 0.001$.
